# Evaluation of Knowledge and Performance of General Dentists in Shiraz, Iran, Regarding Antibiotic Prescription in Endodontic Treatments (2024) 

**DOI:** 10.30476/dentjods.2025.105820.2614

**Published:** 2026-06-01

**Authors:** Mobarakeh Nazari, Maryam Bakhtiar, Fahime Alimardani, Seyed Ali Mirhoseini Sarvestani, Bahar Asheghi

**Affiliations:** 1 Dentist, Shiraz University of Medical Sciences, Shiraz, Iran.; 2 Oral and Dental Disease Research Centre, Dept. of Dental Public Health, School of Dentistry, Shiraz University of Medical Sciences, Shiraz, Iran.; 3 Postgraduate Student, Dept. of Endodontic, School of Dentistry, Shiraz University of Medical Sciences, Shiraz, Iran.; 4 Dept. of Endodontic, School of Dentistry, Shiraz University of Medical Sciences, Shiraz, Iran.

**Keywords:** Antibiotics, Antibiotic Resistance, Necrosis, Dental Pulp, Periapical Disease, Root Canal Therapies

## Abstract

**Background:**

Inappropriate antibiotic prescribing in dentistry contributes significantly to global antibiotic resistance. Clinical guidelines published by the American association of endodontists (AAE) explicitly state that clinicians should avoid prescribing antibiotics for uncomplicated dentoalveolar infections unless systemic symptoms are present. Evaluation of compliance with current antibiotic prescribing guidelines and identifying the gaps between knowledge and clinical performance of clinical practitioners is essential.

**Purpose:**

This study was conducted to assess the knowledge and clinical performance regarding antibiotic use in endodontic treatments among general dentists in Shiraz, Iran, regarding the latest AAE guidelines.

**Materials and Method:**

A cross-sectional study was conducted using a validated questionnaire (Content Validity Index= 0.89, Cronbach’s α= 0.82) distributed to 140 general dentists and residents in Shiraz via random sampling. The questionnaire assessed (1) demographic characteristics, (2) knowledge of current guidelines, and (3) clinical prescribing performance through case scenarios. We analyzed data from 103 completed responses using IBM SPSS v27.0 (IBM Corp., Armonk, NY, USA) with descriptive statistics, chi-square tests, and binary logistic regression (*p* Value < 0.05).

**Results:**

While participants demonstrated moderate to good theoretical knowledge (70.9% correctly identified first-line antibiotics), significant gaps existed in clinical application. Notably, 69.9% of participants prescribed antibiotics inappropriately for immunocompromised patients. Residents exhibited significantly better practical guideline compliance than general practitioners (*p*= 0.019). No significant associations were found with gender, clinical experience, or workplace. Also, 88.3% expressed need for further training.

**Conclusion:**

Considering moderate to good knowledge, coupled with a low to moderate performance level, significant deviations from AAE guidelines highlight the need for targeted educational interventions, including workshops and curriculum integration, to improve antibiotic stewardship.

## Introduction

Various bacteria inhabit the oral cavity. When the epithelial barrier is compromised, these bacteria may enter the bloodstream, potentially causing systemic infections such as bacterial endocarditis [ 1 ]. Antibiotics used appropriately can reduce the duration of bacterial infection and its consequences, such as infection spreading to adjacent tissues or systemic involvement. Nevertheless, various conditions call for the prescription of antibiotics. 

Antibiotics are the most frequently prescribed medications in dentistry [ 2 ]. Substantial evidence demonstrated a significant correlation between antibiotic use and increasing antimicrobial resistance, particularly in regions with high antibiotic consumption compared to areas with lower usage rates [ 3 ].

According to the World Health Organization (WHO), the emergence of increased antibiotic resistance is a major global health challenge. Moreover, they estimate that bacterial antimicrobial resistance was directly responsible for 1.27 million global deaths in 2019 and contributed to 4.95 million deaths [ 4 ]. The multifactorial threats of antimicrobial resistance have resulted in different complex issues affecting countries across the globe. It affects not only patients, but also the healthcare system and the economy [ 5 ].

While antimicrobial prescribing has received significant attention in the medical literature [ 6 - 7 ], the dental public health community still needs to pay more attention to antimicrobial stewardship [ 8 ].

Clinical guidelines published by the American association of endodontists (AAE) explicitly state that clinicians should avoid prescribing antibiotics for uncomplicated dentoalveolar infections unless systemic symptoms are present [ 9 ]. Despite guidelines emphasizing operative interventions, studies report overuse of antibiotics in routine dental care [ 10 ]. Dentists frequently prescribe antibiotics even in the absence of clinical signs of infection and when no concomitant local treatments are required [ 11 - 13 ]. Multiple national studies reveal significant disparities in dentists' awareness of clinical indications for antibiotic use [ 14 - 16 ].

To the best of our knowledge, comprehensive data on dentists' adherence to AAE guidelines regarding antibiotic use remains limited, necessitating further investigations. Given that clinical guidelines are regularly updated, ongoing research is essential to monitor compliance with the latest recommendations. To our knowledge, no prior study has assessed guideline adherence among general dentists in Shiraz in 2024. Concerning the dentists' role in antimicrobial resistance through inappropriate prescriptions, the present study aimed to evaluate the knowledge and performance of general dentists in Shiraz regarding antibiotic prescription according to the latest guidelines for endodontic dental treatments in 2024. 

## Materials and Method

Researchers developed a questionnaire (Appendix 1) to evaluate dentists' guideline-based knowledge and performance in endodontic antibiotic prescribing.

**Appendix 1:**
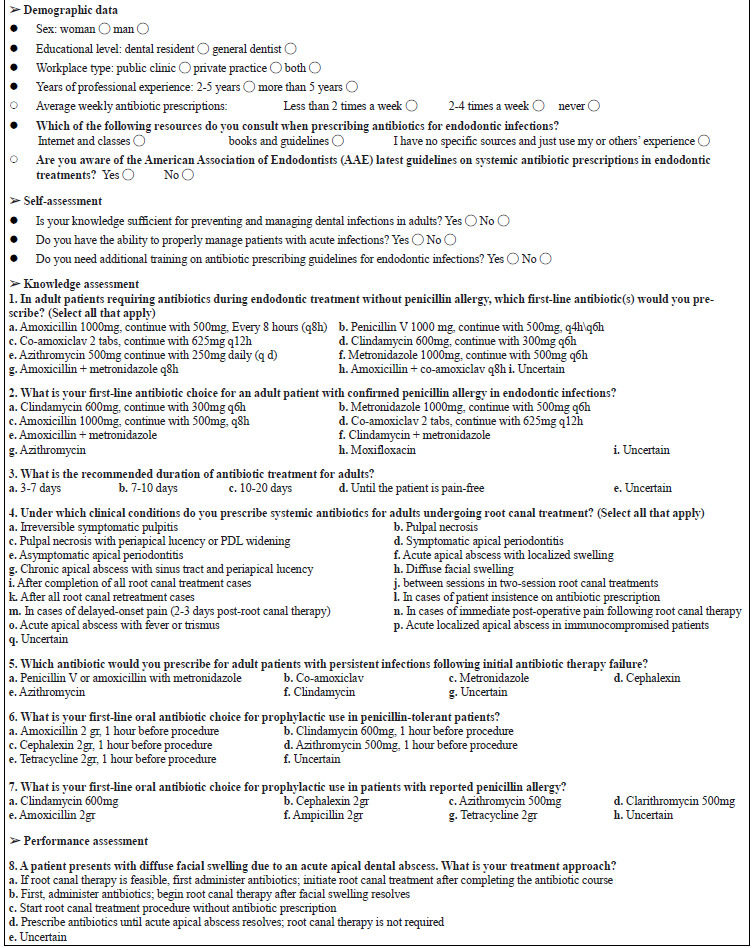
Questionnaire

The questionnaire was designed based on AAE guidelines (2017) and peer-reviewed published literature, validated by three board-certified endodontists (Content Validity Index/ CVI=0.89), with internal consistency confirmed via Cronbach’s alpha (α=0.82). Inclusion criteria comprised general dentists and residents practicing in Shiraz with at least 2-5 years of endodontic experience and signed informed consent, while exclusion criteria included specialists or non-practicing clinicians; questionnaires demonstrating non-engagement (e.g., uniform responses) were excluded. A pilot study with 20 general dentists (excluded from the main study) confirmed clarity, and no revisions were made. The study employed simple random sampling to recruit 140 general dental practitioners from public and private healthcare facilities. We calculated the sample size using parameters from comparable field studies (d=5%, α=0.05, power=80%), resulting in 103 participants. After pre-study communication (explaining objectives and obtaining consent), participants completed the questionnaire, which covered: (1) demographic data (gender, experience, workplace), (2) self-assessment, (3) knowledge of antibiotic prescription principles, and (4) clinical case-based performance (treatment plans for endodontic cases). For each question, participants were required to select one answer; in cases where multiple correct answers were possible, this was explicitly stated in the question stem. Of 140 invited dentists accounting for non-responses, 103 met inclusion criteria (response rate=73.6%).

Regarding the scoring method, the questionnaire was scored by assigning one point to each correct answer (incorrect/unknown responses= 0). Multi-option questions were scored additively, with each correct sub-item contributing to the question’s total. Final scores were calculated as the sum of all item scores, ranging from 0 to 14.

Participants who answered fewer than half of the questions correctly were classified as having weak knowledge or performance; those who answered half of the questions correctly were considered to have a moderate level; participants with more than half of the questions answered correctly were categorized as having good knowledge or performance; and those who answered all questions correctly were regarded as having an excellent level.

### Ethical Considerations

Ethical approval was obtained from Shiraz University of Medical Sciences (I.R.SUMS.DENTAL.REC.1402.005). Participants provided written informed consent. Data were anonymized to ensure confidentiality.

### Statistical Analysis

Data analysis was initially performed using IBM SPSS Statistics version 22.0 due to institutional availability at the time of the study. Following the reviewer’s valuable suggestion, key analyses were re-conducted using IBM SPSS Statistics version 27 to ensure the robustness and accuracy of the results. The findings were consistent across both versions, confirming the reliability of the statistical outcomes. Descriptive statistics, including frequencies, means, and standard deviations, were calculated. Non-parametric tests such as Spearman’s correlation and Kruskal-Wallis were applied to assess relationships between variables. Associations between knowledge, performance, and professional qualification (general dentists vs. residents) were evaluated using chi-square tests and binary logistic regression, with responses dichotomized as correct or incorrect. Statistical significance was set at *p*< 0.05.

## Results

The study included 140 general dentists and residents studying in various specialty courses. 103 of the 140 questionnaires given to the participants were correctly completed.

The findings of the current study demonstrated that dentists' knowledge of antibiotic prescription principles in endodontic treatments was moderate to good, while their performance in evidence-based prescribing ranged from low to moderate.

No significant correlation was observed between participants' knowledge and their clinical performance. Participants included 70 general dentists (67.9%) and 33 residents (32.1%). Residents’ performance scores were significantly higher (*p*= 0.019), likely due to academic supervision during training. Of the 103 respondents, 68 (66%) were women and 35 (34%) were men. In terms of experience, 73.8% had 2-5 years of clinical practice, while the remaining 26.2% had over five years. Antibiotic prescribing frequency varied; 44.7% reported prescribing less than twice a week, 31% two to four times weekly, and 24.3% never prescribed antibiotics.

When asked about their sources of information for antibiotic prescribing, 51.4% of participants reported using guidelines and textbooks, 36% relied on no specific references, and 12.6% utilized continuing education courses and online resources.

In terms of workplace distribution, 63.1% worked in public healthcare facilities, 29.2% in private practices, and 7.7% in both sectors.

Eighty-seven participants (84.5% of the sample) demonstrated no awareness of current antibiotic prescription guidelines, while only 16 (15.5%) reported familiarity with updated protocols. Analysis of demographic variables revealed no statistically significant correlation between participant characteristics and their knowledge of guidelines or clinical performance levels. Notably, only residents demonstrated significantly better adherence to antibiotic guidelines compared to general dentists.

In the self-assessment section of the questionnaire, participants were asked to evaluate their own knowledge and performance through three key questions: (1) whether they considered their knowledge of preventing and managing adult dental infections sufficient, (2) whether they felt confident in managing acute dental infections appropriately, and (3) whether they perceived a need for further education in this domain. Statistical analysis revealed no significant correlation between participants' self-reported responses and their actual knowledge or clinical performance levels (*p*> 0.05).

In the knowledge assessment section, dentists were asked to identify their first-line antibiotic choice for non-penicillin-allergic patients. Amoxicillin was the most frequently reported option (35.9%). For penicillin-allergic patients, clindamycin was selected by the majority (50.5%). Among the 103 participating dentists, 50.5% reported recommending an antibiotic duration of 7-10 days, while 43.7% considered 3-7 days as the appropriate duration.Dentists were asked whether they would prescribe antibiotics in the listed hypothetical clinical scenarios. The results are presented in Table 1.

**Table 1:** Indications for prescribing antibiotics

Clinical state		Yes (%)	No (%)
A.	Irreversible symptomatic pulpitis	1(1%)	102(99%)
B.	Pulpal necrosis	10(9.7%)	93(90.3%)
C.	Pulpal necrosis with radiolucency or periodontal ligament widening	13(12.6%)	90(87.4%)
D.	Symptomatic apical periodontitis	8(7.8%)	95(92.2%)
E.	Non-symptomatic apical periodontitis	2(1.9%)	101(98.1%)
F.	Acute apical abscess with localized swelling	53(51.5%)	50(48.5%)
G.	Chronic apical abscess with sinus tract and apical radiolucency	21(20.4%)	82(79.6%)
H.	Diffuse facial swelling	73(70.9%)	30(29.1%)
I.	After all root canal treatments	0(0%)	103(100%)
J.	Between two sessions	6(5.8%)	97(94.2%)
K.	After all retreatments	5(4.9%)	98(95.1%)
L.	When patients insist	1(1%)	102(99%)
M.	When the pain starts 2 to 3 days after root canal treatments	18(17.5%)	85(82.5%)
N.	When the patient feels pain exactly after root canal treatments	2(1.9%)	101(98.1%)
O.	Acute apical abscess with fever or trismus	80(77.7%)	23(22.3%)
P.	Acute localized apical abscess in immunocompromised patients	73(70.9%)	30(29.1%)
Q.	No idea	6 (5.8%)	97(94.2%)

We asked dentists to identify their second-line antibiotic choices for non-allergic patients when initial therapy failed. The most frequently reported alternatives were Penicillin V or amoxicillin combined with metronidazole (27.2%), and co-amoxiclav (22.3%). 

For cases requiring prophylactic antibiotics prior to root canal treatment in adults, amoxicillin (2g administered one hour preoperatively) was the first-line choice (90.3%). Concerning penicillin-allergic patients, clindamycin emerged as the most frequently selected alternative (66%).

**Figure 1:**
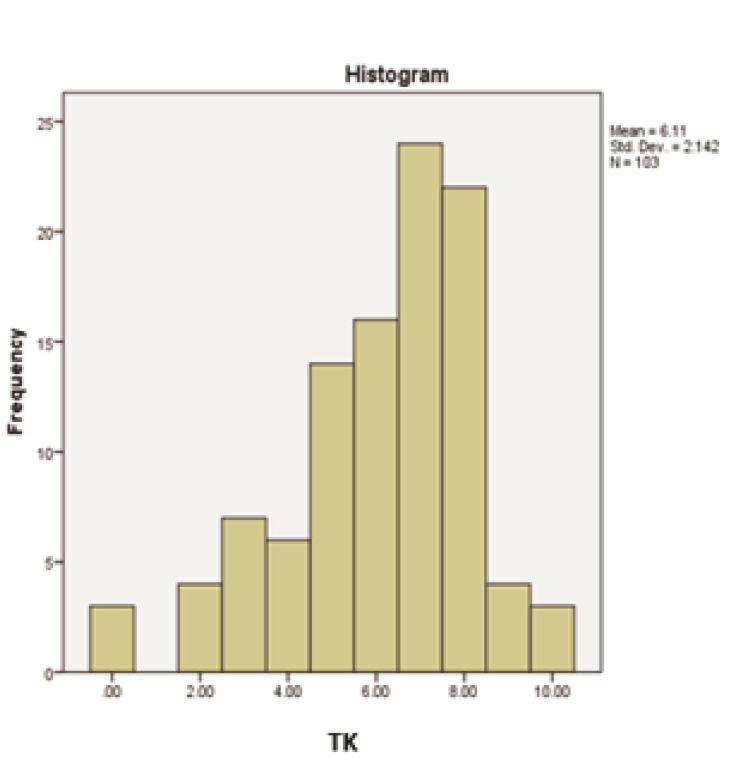
Distribution of respondents’ total knowledge (TK) scores

**Figure 2:**
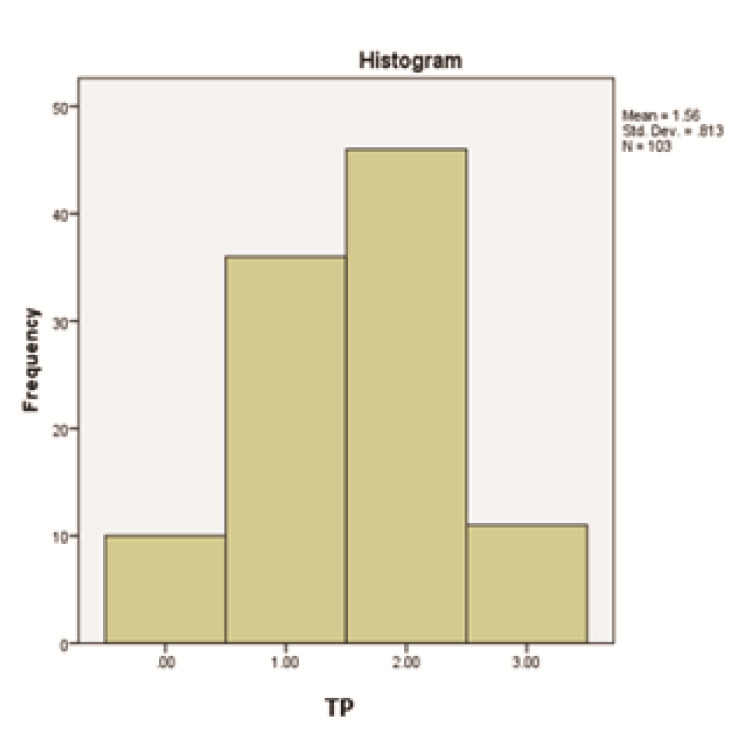
Distribution of respondents’ total performance (TP) scores

The respondents' knowledge and performance metrics are presented in Figures 1 and 2. 

## Discussion

World health organization (WHO) states that the rise of antibiotic resistance is a significant global health issue [ 17 ]. Concerning the dentists' role in antimicrobial resistance through inappropriate prescriptions, the present study aimed to evaluate the knowledge and performance of general dentists in Shiraz regarding antibiotic prescription according to the latest guidelines for endodontic dental treatments in 2024. According to this study's results, dentists’ knowledge of the indications for prescribing antibiotics in endodontic treatments was moderate to good, while their performance was low to moderate. The results obtained from the study of Nabavizadeh et al. [ 20 ] also indicated that knowledge of general dentists in Shiraz needed enhancement. In the study of Zahabiyoun et al. [ 21 ], dentists' knowledge level needed improvement. The study of Rodríguez-Fernández et al. [ 22 ] demonstrated that modifiable factors influenced prescribing quality among dentists in Spain and suggested the development of educational and training programs for dentists.

No significant gender-based differences in antibiotic prescribing knowledge or performance were observed in this study. These findings are consistent with previous research by Moafy *et al*. [ 18 ] in Gorgan and Nabavizadeh *et al*. [ 20 ] in Shiraz, both of which reported similar patterns across genders.

Our findings revealed no significant difference in knowledge levels between residents and general dentists; however, residents' performance was significantly higher. Their superior performance may be linked to their academic engagement, where treatment plans are supervised by faculty members. Such supervision likely enhanced clinical decision-making, leading to more effective treatment plans.

A comparison between dentists with 2-5 years of clinical experience and those with over five years of practice revealed no significant differences in knowledge or clinical performance. The absence of significant differences may reflect both deficiencies in guideline-based training during dental education, leaving even recently graduated dentists without evidence-based competencies, and practitioners' insufficient engagement with updated guidelines. However, in contrast to these findings, a study by Maryami *et al*. [ 19 ] reported an inverse correlation between years of experience and guideline awareness. Similarly, Moafy *et al*. [ 18 ] found that more experienced participants had significantly less awareness of antibiotic prescribing. The study by Moafy *et al*. [ 18 ] also found that more experienced participants had significantly lower awareness of prescribing antibiotics.

 Analysis revealed no significant differences in knowledge or performance scores between participants who never prescribed antibiotics and those who prescribed them less than twice a week or two to four times a week. A potential reason for this finding is that all three groups may need further education on the relevant guidelines and protocols, as the observed performance demonstrated inadequate compliance.

Participants were also asked how they obtained information regarding the correct prescription of antibiotics. Statistical analysis revealed no significant differences in knowledge or performance between dentists who relied on books and guidelines and those who obtained information from the internet, educational courses, or personal and colleagues’ experience. This may be attributed to the necessity for all practitioners to regularly consult the latest guidelines. These findings emphasize the need for up-to-date, evidence-based educational resources and training to optimize antibiotic prescribing performance.

Fewer than half of participants (49.5%) thought their knowledge was sufficient when asked to respond to their self-assessment questions, and 88.3% of respondents felt the need to attend classes and training sessions on this subject, which highlights the need for additional training and educational programs.

Among dentists who responded to the first knowledge-assessment question regarding the first-choice antibiotic for root canal treatments in adults without a penicillin allergy, the most frequently selected response was the combination of amoxicillin and metronidazole (70.9%). In contrast, amoxicillin (35.9%) and penicillin V (10.7%), the recommended first-line antibiotics according to the most recent AAE guidelines, were chosen less often. The preference for the amoxicillin-metronidazole combination as the first choice highlights a significant deviation from current evidence-based recommendations. This discrepancy underscores the need for additional education and training. Amoxicillin was the most prevalent selective antibiotic in the study of Jaunay et al. [ 23 ]. In the study by Mainjot *et al*. [ 12 ], amoxicillin was identified as the most commonly prescribed first-line antibiotic in root canal therapy. As observed in two aforementioned studies, participants demonstrated a higher level of knowledge in selecting appropriate first-line antibiotics for patients without penicillin allergies compared our findings.

For patients with penicillin allergies, 56.3% of respondents selected the three correct antibiotic options-clindamycin, azithromycin, and moxifloxacin. Notably, only 5.8% of dentists identified azithromycin as a correct choice, and none selected moxifloxacin as a correct option. This indicates that while more than half of participants were aware of the appropriate alternatives, the majority selected clindamycin, while azithromycin and moxifloxacin were chosen by very few. These findings suggest a gap in evidence-based knowledge among dentists regarding antibiotic selection for penicillin-allergic patients and highlight the need for improved education in this area.

In the study by Yingling [ 24 ], penicillin V was identified as the first-choice antibiotic for this patient group, followed by amoxicillin. Comparative analysis revealed that dentists in the Yingling *et al*.'s study exhibited a higher level of awareness and adherence to evidence-based prescribing guidelines. This suggests a need for enhanced education and stewardship to improve adherence to guidelines among dentists.

Regarding antibiotic duration, approximately half of the respondents (43.7%) selected a seven-day course, while 50.5% chose a seven- to ten-day regimen. Encouragingly, only a small proportion of dentists selected an incorrect duration of 10 to 20 days, and none reported prescribing antibiotics until the patient’s pain subsided. These findings highlight the need to provide dentists with up-to-date, evidence-based information regarding optimal antibiotic use. Similarly, in the study by Jaunay *et al*. [ 23 ], participants also tended to prescribe lower doses of antibiotics over extended periods.

According to the results of this study, dentists most frequently prescribed antibiotics for acute apical abscess with fever or trismus, localized apical abscess in immunocompromised patients, and cases of diffuse facial swelling. All of these are indications for antibiotic prescription. Furthermore, none of the participants prescribed antibiotics postoperatively in any root canal treatments. These results indicate good knowledge among the participants.

Notably, latest AAE guidelines recommend antibiotic prescriptions when pain develops two to three days post-procedure, yet only a minimal proportion of respondents demonstrated awareness of this evidence-based indication.

Furthermore, more than half of dentists prescribed antibiotics for acute apical abscesses with localized swelling, which did not comply with the guidelines and is an unnecessary prescription. 

Based on the distribution of responses, most participants reported that they would prescribe antibiotics in clinical scenarios involving the presence of an abscess. However, current guidelines indicate that antibiotic therapy is generally not recommended for abscesses unless systemic manifestations such as fever, trismus, or immunocompromised status are present. This finding highlights a discrepancy between clinical practice and evidence-based recommendations, as antibiotics should be reserved for cases with systemic involvement or in patients at greater risk of complications.

If the first antibiotic was not effective, the most common second-line choices were penicillin V / amoxicillin-metronidazole (27.2%), co-amoxiclav (22.3%), cefalexin (8.7%), and clindamycin (8.7%). While cefalexin use deviates from guidelines, other selections align with recommendations, reflecting strong guideline awareness among general dental practitioners in Shiraz, though further education is needed.

For prophylactic antibiotics in non-allergic patients, 90.3% of respondents chose amoxicillin. This choice aligns with the guidelines and reflects a good level of respondents’ knowledge. However, given that 7.8% of participants lacked awareness of the correct protocol and others selected non-guideline-compliant options.

Furthermore, in a systematic review conducted by Cuevas-Gonzalez *et al*. [ 25 ], the appropriateness of prescribing prophylactic antibiotics prior to various oral surgical procedures was evaluated. The review found that beta-lactam antibiotics were the most commonly prescribed agents among dentists across all included studies, with amoxicillin being the predominant drug in more than half of the studies reviewed. These findings are consistent with the current study, indicating that amoxicillin- and more generally, beta-lactams- are the primary antibiotics prescribed for prophylactic purposes in both surgical and endodontic treatments.

When participants were asked about their choice of prophylactic antibiotic in patients with penicillin allergy, their selections demonstrated a high level of concordance with established guidelines. Conversely, the study by Nabavizadeh *et al*. [ 20 ] found that only a small percentage of dentists in Shiraz were aware of all indications for prophylactic antibiotic prescription, and overall awareness regarding these indications was assessed as low. In summary, although dentists’ knowledge regarding the indications for prophylactic antibiotic use appears limited, their knowledge of the correct prophylactic antibiotic selection is relatively high.

To evaluate dentists’ performance, participants were first asked to describe their management approach for a patient presenting with diffuse facial swelling secondary to an acute apical abscess. Notably, 41.7% of respondents reported prescribing antibiotics prior to initiating root canal therapy when immediate treatment was feasible. However, current clinical guidelines explicitly state that antibiotics are unnecessary and root canal treatment should not be delayed if definitive dental intervention can be performed. Consequently, antibiotic prescribing patterns in such scenarios require realignment with evidence-based protocols to mitigate inappropriate antimicrobial use.

Participants were also asked to describe their management approach for a patient presenting with severe pain during mastication and upon consuming cold beverages, persisting for one hour. Notably, 84.5% of respondents appropriately selected root canal treatment without antibiotic prescription, which aligns with evidence-based guidelines. This observation highlights that clinicians possess a strong understanding of pulpal and peri-radicular pathologies, which likely contributes to more guideline-compliant treatment decisions in such clinical scenarios.

Participants were additionally asked to outline their treatment approach for the aforementioned patient with immunodeficiency. In these cases, 30.1% of respondents adhered to evidence-based guidelines by selecting root canal therapy without antibiotic prescription. Conversely, 69.9% prescribed antibiotics, reflecting suboptimal adherence to antimicrobial stewardship principles in the management of immunocompromised patients.

One of the limitations of this study was the potential for response bias, as the data were collected through a self-reported questionnaire. Dentists may have overestimated their compliance with recommended practices, either consciously or unconsciously, leading to an inflation of reported adherence rates. This type of bias is common in survey-based research and should be considered when interpreting the results. 

Given the global rise in antibiotic resistance and demonstrated gaps in dentists' knowledge and performance to prescribing guidelines, addressing these challenges necessitates a dual approach. First, implementing interactive, case-based workshops aligned with AAE guidelines for dentists is critical to improving antibiotic selection and duration. Second, integrating antimicrobial stewardship principles into dental curricula is essential to promote evidence-based decision-making. These interventions have the potential to reduce inappropriate prescribing, enhance patient outcomes, and curb resistance rates. However, the reliance on self-reported data in this study, which risks overestimating adherence due to response bias, underscores the need for cautious interpretation. Future research should employ mixed-method approaches to validate prescribing behaviors and assess the long-term efficacy of educational reforms. 

## Conclusion

This study highlighted a critical gap between knowledge and practice in antibiotic prescribing among dentists and residents in Shiraz. While 70.9% correctly identified first-line antibiotics, 69.9% inappropriately prescribed them for immunocompromised patients. Residents outperformed general practitioners, underscoring the value of academic training. Major concerns include unnecessary prescriptions for localized infections and frequent use of non-guideline antibiotic combinations. With 88.3% of dentists requesting further education, targeted interventions like mandatory workshops and curriculum reforms are urgently needed to improve adherence to guidelines and combat antibiotic resistance. Addressing these issues through structured stewardship programs could significantly enhance clinical practice and public health outcomes.
